# Conformational spread drives the evolution of the calcium–calmodulin protein kinase II

**DOI:** 10.1038/s41598-022-12090-y

**Published:** 2022-05-19

**Authors:** Shahid Khan

**Affiliations:** 1 Molecular Biology Consortium, Lawrence Berkeley National Laboratory, Berkeley, CA 94720 USA; 2 SBA School of Science and Engineering, LUMS, Lahore, Pakistan; 3 Laboratory of Cell Biology, NINDS, NIH, Bethesda, MD 20892 USA

**Keywords:** Computational biology and bioinformatics, Neuroscience, Structural biology

## Abstract

The calcium calmodulin (Ca^2+^/CaM) dependent protein kinase II (CaMKII) decodes Ca^2+^ frequency oscillations. The CaMKIIα isoform is predominantly expressed in the brain and has a central role in learning. I matched residue and organismal evolution with collective motions deduced from the atomic structure of the human CaMKIIα holoenzyme to learn how its ring architecture abets function. Protein dynamic simulations showed its peripheral kinase domains (KDs) are conformationally coupled via lateral spread along the central hub. The underlying β-sheet motions in the hub or association domain (AD) were deconvolved into dynamic couplings based on mutual information. They mapped onto a coevolved residue network to partition the AD into two distinct sectors. A second, energetically stressed sector was added to ancient bacterial enzyme dimers for assembly of the ringed hub. The continued evolution of the holoenzyme after AD–KD fusion targeted the sector’s ring contacts coupled to the KD. Among isoforms, the α isoform emerged last and, it alone, mutated rapidly after the poikilotherm–homeotherm jump to match the evolution of memory. The correlation between dynamics and evolution of the CaMKII AD argues single residue substitutions fine-tune hub conformational spread. The fine-tuning could increase CaMKIIα Ca^2+^ frequency response range for complex learning functions.

## Introduction

The frequency decoding of calcium pulses by calcium calmodulin-dependent kinase II (CaMKII) is central to CaMKII control of memory in the brain^1^. Remarkably, individual holoenzymes decode Ca^2+^ pulses^2^. The multi-subunit holoenzyme architecture, a two-ring stack of subunits with mirror symmetry^3^, is unique among members of the large calmodulin-dependent kinase (CaMK) family. Recent reviews have detailed the relationship of CaMKII to the CaMK family^4^ and its role in synaptic function^4,5^.

In all CaMKII isoforms (α,β,γ,δ), individual subunits consist of a canonical kinase domain (KD) with a C-terminal pseudo-substrate regulatory segment adjacent to a CaM-binding domain (R) connected via flexible linkers to an association domain (AD) that forms the central hub. The Ca^2+^/CaM triggered dissociation and capture of R by an adjacent KD enables trans-phosphorylation of R residue T286 in CaMKIIα (T287 in other isoforms). Activation by threonine trans-phosphorylation is common to all isoforms. CaMKIIα is the dominant isoform in the brain where αβ heterooligomers form by expression of the β isoform. Single polymorphisms in the human α and β isoforms result in intellectual disabilities^6–8^. Activation by T286 trans-phosphorylation occurs in CaMKII dimer constructs as well as in holoenzymes^9,10^. The study of isoform variability has focused on the alternatively spliced KD–AD linkers that modulate tissue localization, interactions with the actin cytoskeleton and the balance between activating and inhibitory phosphorylation^11–16^. Variations in the conserved AD responsible for ring assembly were not as well analyzed until recently. We now know that kinase activity is modulated by endogenous ligands that bind to the hub^17^ and determined by differences between α and β isoform hubs as established by chimeric constructs^18^. Four of the six CaMKIIα variants found in patients with schizophrenia localized to the AD^8^. A CaMKIIβ hub deletion has revealed isoform-specific differences in holoenzyme formation^19^. Finally, human CaMKIIα R peptides interact with and destabilize their hubs^20^, consistent with activation dependent subunit exchange^21,22^. Here, motivated by these studies, I have analyzed hub dynamics and evolution to evaluate the importance of the ringed architecture for CaMKII function.

An atomic structure of the human CaMKIIα holoenzyme is available, together with crystal structures of the CaMKII AD superfamily from many microbial and metazoan species. In addition, many amino acid sequences from this family have been deposited. I used a sub-assembly of the CaMKIIα holoenzyme to generate a conformational ensemble in silico. I analyzed this ensemble to map the long-range conformational spread between KDs via the AD hub. Residue contacts were analyzed to determine energetically stressed sectors. The increased energetic cost is compensated by enhanced function, typically binding propensity^23^, as illustrated by a protein databank survey that reported stabilization of energetically stressed contacts clustered near protein–protein association interfaces by complex formation^24^. Multiple sequence alignment (MSA) of the CaMKII AD sequences identified coevolved residue contacts also shown to be important for function^25^. The most strongly coevolved contacts localized to the AD interfaces. I further compared phylogeny based on the MSA with that based on crystal structures and sensory behavior. I found that, first, variations of eukaryotic sequences most closely related to the human CaMKIIα AD mapped to the lateral ring contacts rather than the primordial dimer contact that was targeted in the bacterial sequences. Second, the behavioral phylogeny based on learning assays correlated with the sequence phylogeny behavior of the α, but not the other isoforms. I conclude that the AD hub propagates lateral conformational spread, based on the dynamics, and that the residue contacts that mediate this spread are important for function, based on the energetics and evolution. I propose that the distinct evolutionary trajectory of the α isoform reflects tuning of conformational spread in the ringed holoenzyme to extend the CaMKII response range to Ca^2+^ pulses for complex learning tasks in the brain.

## Results

### Hub β-sheet dynamics mediate long-range KD–KD coupling

**Figure 1:**
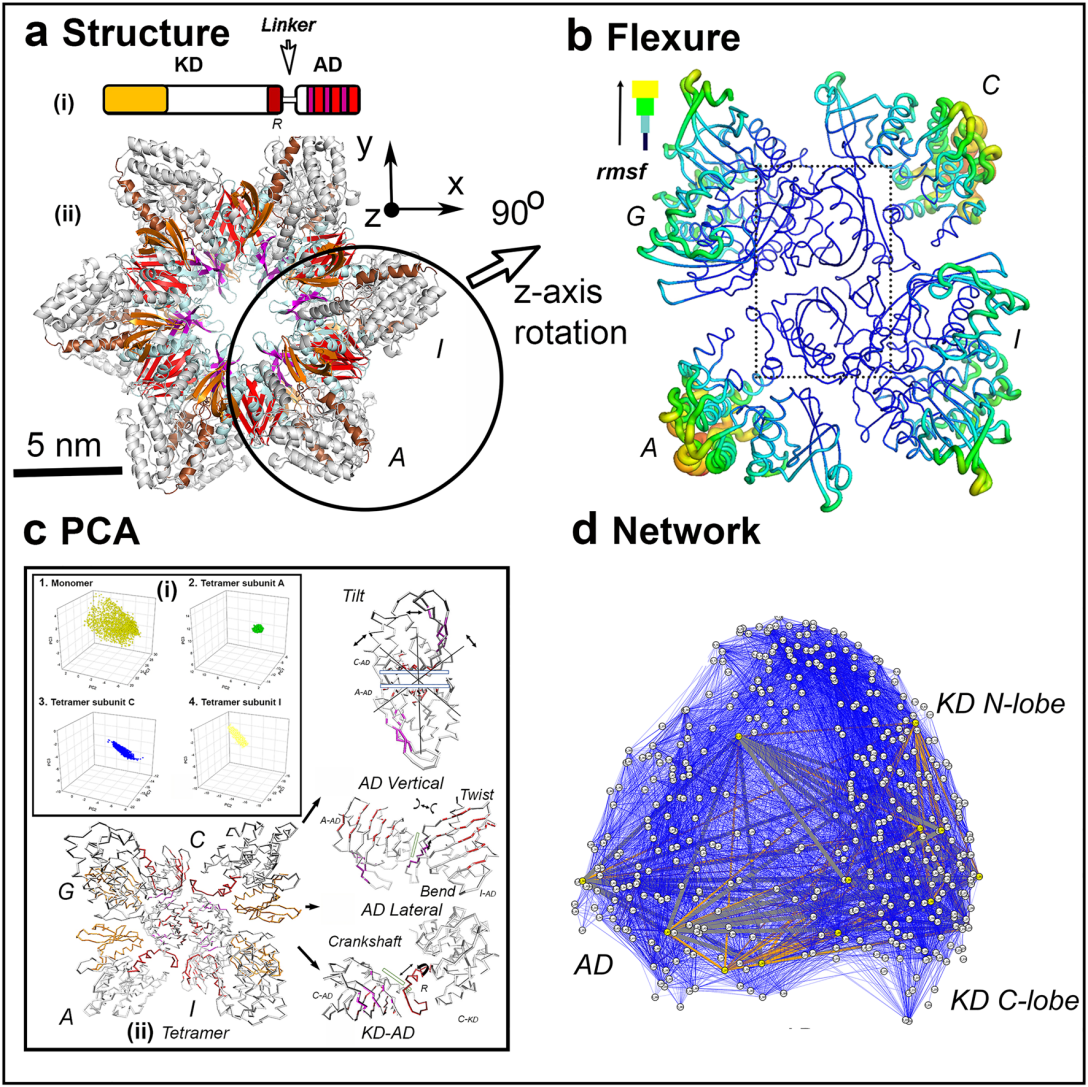
(**a**) Architecture. (i) Subunit. Disordered linkers with varying lengths and composition connect the kinase domain (KD (N-lobe (orange), C-lobe (white)) with the association domain (AD). The pseudo-substrate, regulatory segment (R (brown) binds Ca^2+^/CaM. The AD β-sheet forms vertical (red) and lateral (magenta) ring contacts. (ii) Assembly. The ADs form the central hub in the multi-subunit holoenzyme (CaMKIIα 3SOA.PDB). A tetramer (circle) was extracted for analysis of conformational fluctuations. (**b**) Flexure. The flexibility (rmsf)) profile derived from the tetramer conformational ensemble. The tetramer orientation shown is rotated by 90° relative to the orientation in panel a (Supplementary Video S1). (**c**) Principal Component Analysis (PCA). (i). PCIPC2PC3 plots of the human CaMKIIα AD; (ii) A conformation in the CaMKIIα tetramer tCONCOORD ensemble (Supplementary Video S2) used for PCA, with PCs 1–3 mapped onto the key contacts (AD Vertical (Vert-Dim), Tilt. AD Lateral (Lat-Dim), Bend and twist. KD–AD. Crankshaft (extension + rotation)). Rectangles represent β sheet long axes. (**d**) Monomer network. Nodes = Residues (Circles). Edges = Dynamic couplings between 4-residue fragments (lines color-coded according to nMI score (high (orange) → low (blue)). Source listed in Table S1.

The crystal structure of the two-stack human CaMKIIα holoenzyme has six subunits per stack (3SOA.PDB). The AD has distinct intra-stack and inter-stack contacts. The holoenzyme may be partitioned into three tetramers that each contain both contacts. In the tetramer ACGI shown in Fig. 1a, subunits A, C form the inter-stack dimer (Vert-Dim) contact. The (A, I) and (C, G) lateral dimers (Lat-Dim) form intra-stack contacts. The KDs also contact the ADs. Intra-subunit (“clip”) contacts of the KD CaM-binding domain with its AD and inter-subunit (“spur”) contacts of the KD DFG activation loop with the AD of the adjacent lateral subunit are seen in the autoinhibited holoenzyme^26^. Conformational ensembles were generated from the ACGI tetramer with tCONCOORD^27^. In brief, tCONCOORD generates another conformer from the tetramer crystal structure by random displacement of its atoms within limits, followed by iterative correction to eliminate bond violations until all bonding constraints are satisfied and a new structure is obtained (see “Methods” (Protein Dynamics) for parameters). The process is then repeated until an ensemble of the desired size is obtained. The tetramer root-mean-square fluctuation (rmsf) profile reported the mean fluctuations for the C^α^ atoms relative to the reference crystal structure obtained for this tCONCOORD ensemble. It was evident from the rmsf profile that small fluctuations of the hub AD domains were coupled to large motions of the associated KD’s (Fig. 1b). The C^α^ atoms of the residue contacts at the Vert-Dim interfacial hinge were more rigid (0.29 ± 0.002 nm) than contacts for the Lat-Dim hinge (0.355 ± 0.016 nm) or the AD–KD “clip” (0.292 ± 0.005 nm) consistent with previous work^21,28^.

I used principal component analysis (PCA) to determine the extent and nature of the long-range collective motions (Fig. 1c, Supplementary Video S1). These motions were encoded by the first three principal components (PCs) (“Methods” (Protein Dynamics)) that constituted a large fraction (0.7 = 0.46(PC1) + 0.14 (PC2) + 0.10 (PC3)) of all motions measured for the tetramer tCONCOORD ensemble. First, I estimated constraints due to the formation of the tetramer on the motion of the component subunits. A tCONCOORD ensemble for a monomer extracted from the holoenzyme was generated and similarly analyzed. The PC1PC2PC3 plots showed that the tetramer subunit motions had reduced amplitude, but increased anisotropy relative to the monomer. The monomer had a reduced PC1 fraction (0.34). Second, I used GROMACS geometric functions to map the PC motions from the tetramer ensemble onto the key contacts. These consisted of orthogonal tilt motions at the Vert-Dim contact and anisotropic β_2_–β_5_ sheet bending and twisting motions at the Lat-Dim contacts. The central hinge was the more rigid Vert-Dim contact rather than the weaker Lat-Dim contact that primarily transmitted the hub β_-_sheet deformations to adjacent subunits. The principal PC1–PC3 components coupled KD–KD twist and rotations. The crankshaft (rotation + extension) KD motion is coupled to the AD–AD β_2_–β_6_ sheet bending and twisting modes. The measurements taken together reveal the KD–AD-KD coupling mechanism as detailed in Supplementary Fig. S1.

I constructed mutual-information (nMI) based networks to encode the local fragment dynamics of local fragments as 1D-strings for comparison with the molecular evolution metrics derived from the MSAs (Fig. 1d). The network analysis revealed how AD flexibility is constrained in the tetramer relative to the free monomer. The DFG α_3_–β_2_ loop was the central peak common to the network centrality profiles of the monomer and the tetramer KDs. The R T286 fragment (R_286_) is the central node in the monomer but is suppressed in the tetramer; a possible consequence of the inter-subunit clip KD–AD contact at the Ca^2+^/CaM binding site located at the other end of the R helix (R_300–306_). The spur KD–AD contact transmits AD motions to rotation-translation of KD helices α_6–7,9_. In the tetramer, the Vert-Dim had prominent β_2_, β_3_ and β_6_ peaks while the Lat-Dim had prominent peaks for the α_1_–α_2_ loop, α_3_ and β_4_–β_5_ junction in the centrality plots consistent with the top network couplings (Supplementary Fig. S1).

### The dynamic network reflects hub residue coevolution

I used two tools to correlate local dynamics with molecular evolution (“Methods” (Structure Analysis)). First, I assessed the energetic cost of residue contacts to diagnose metastable binding surfaces as noted in the Introduction. The frustration score (ΔE_fr_) reported the stabilization energy of the native contact relative to all possible contacts. It was used to partitions contacts into sub-populations with low (“relaxed”) or high (“stressed”) cost energetics. Second, I scored residue coevolution. The score (${S}_{s}$) represented the evolutionary coupling strength between two residue positions, again indicative of a functionally important contact.

**Figure 2:**
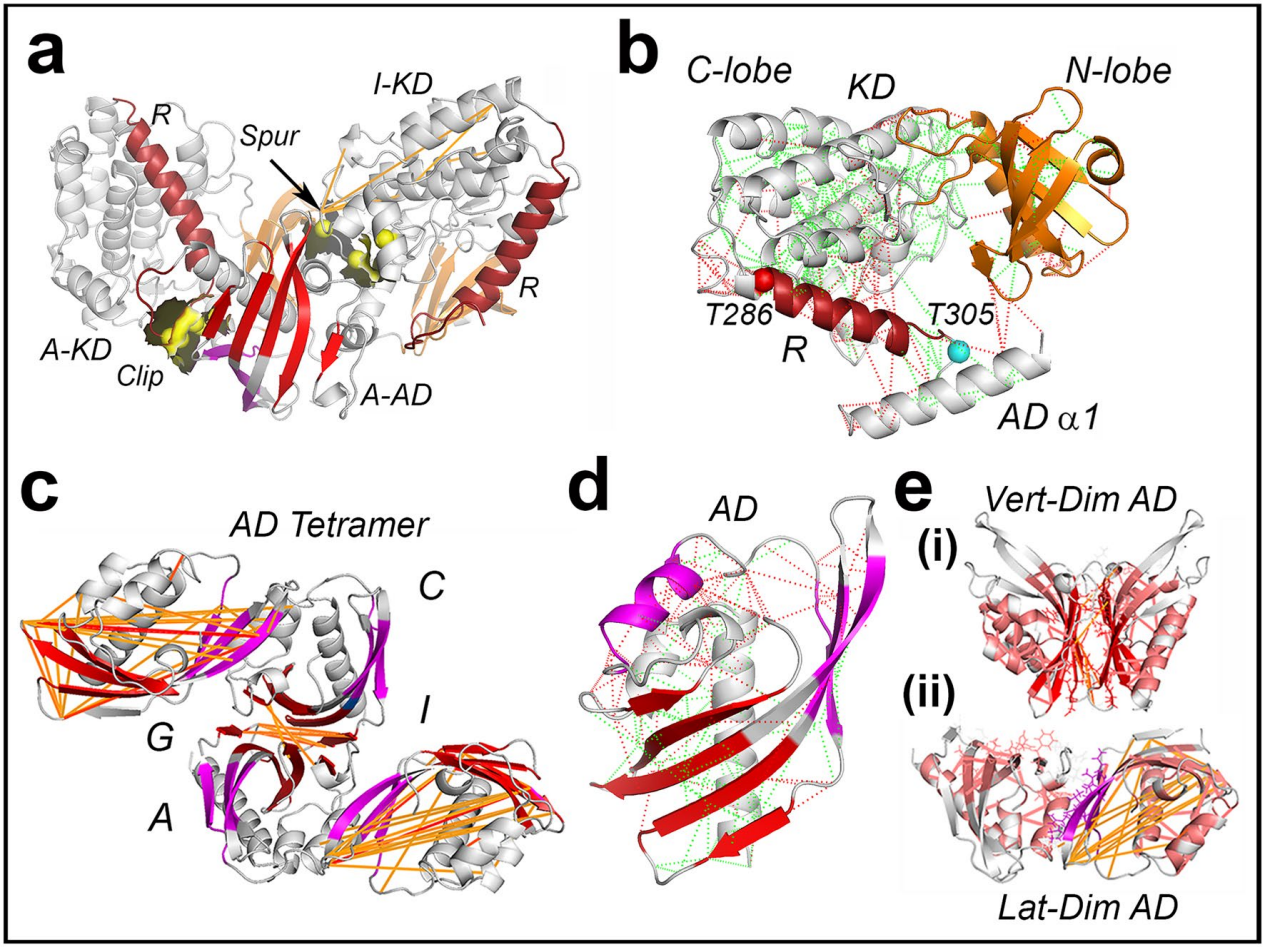
The dynamics and evolution of the CaMKII AD. Hub interfaces and R helix are color-coded as in Fig. 1a. (**a**,**b**). KD–AD Contact. (**a**). Dynamics. The top dynamic couplings computed between 4-residue fragments (yellow (weak) → orange → red (strong)). The KD–AD contact residues and surface (yellow). (**b**) Energetics. Energy frustration—(relaxed (green), stressed (red)). Spheres denote T286 (red), T305 (cyan). (**c**,**d**) AD Fold. (**c**) Interfacial dynamics. ACGI AD tetramer. The dynamic couplings span the interfaces (Vert-Dim (red), Lat-Dim (purple). Supplementary Video S3). (**d**) Energetics. Energy frustration scores are color coded as in B. (**e**) Residue Coevolution. The superposition of the dynamic (thin orange lines) and coevolved (thick salmon lines) couplings adjacent to the (i) Vert-Dim and (ii) Lat-Dim contacts (stick sidechains). 3D-views in Supplementary Videos S4–S7. Source listed in Table S1.

The spur KD–AD contact coupled AD fluctuations to α-helices in the KD C-lobe (Fig. 2a). The contacts made by the regulatory segment R with both the KD C-lobe and AD were energetically stressed based on the ΔE_fr_ scores (Fig. 2b). Interfacial couplings that propagated across and along the two stacks dominated the AD dynamic network. The Vert-Dim dynamic couplings connected the β_2_–β_4_ sheet center in one AD with the α_2_–β_1_ and β_3_–β_4_ loops in the other AD across the rigid (β_3_–β_4,_ β_6_ strands, α_2_–α_3_ loop) contact. The top (1%) dynamic couplings at the Lat-Dim contact of helix α_3_ with the adjacent β_3_–β_5_ loops, changed AD β-sheet curvature (Fig. 2c). The ΔE_fr_ profiles revealed the energetic cost of the evolution of CaMKIIα Lat-Dim contact (Fig. 2d). Its structural elements formed stressed interactions relative to the relaxed contacts associated with the Vert-Dim interface. The strongly coevolved residue contacts scaffolded the dynamic couplings as seen in maps of the Vert-Dim and Lat-Dim dimer complexes. The contacts formed two sectors—the larger sector stitched helices α_2–3_ with strands β_1–3_ at the Lat-Dim contact, while the smaller one bonded the central β_2–5_ sheet at the Vert-Dim contact with long helix α_1_ (Fig. 2e).

**Figure 3:**
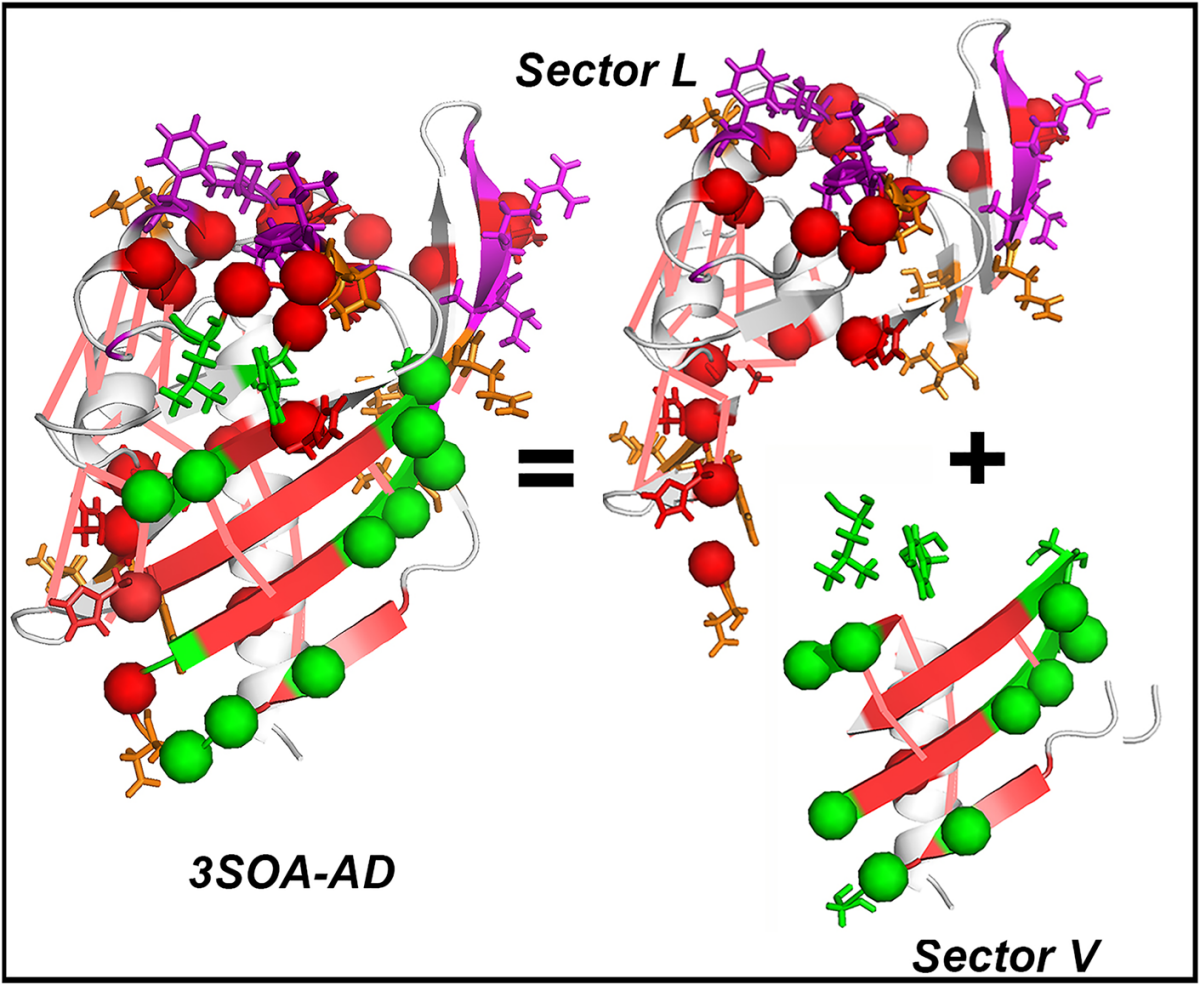
The two sectors of the CaMKIIα AD. The coevolved residue network of the primordial vertical dimer (Sector V) has energetically relaxed residues (green spheres) at the Vert-Dim contact interface (red β strands). The coevolved residue network (thick salmon lines) of the Lat-Dim contact (Sector L) has energetically stressed residues (red spheres) at the contact interface (purple α-helix, β strands). Stick representations denote residues at the interface (purple, green, red) or part of adjacent dynamic couplings (gold). 3D-view in Supplementary Video S8. Source listed in Table S1.

High-throughput genome sequencing and X-ray crystallography^29,30^ identified the CaMKII-AD superfamily, sometimes cited as the NTF2 superfamily (PF08332) due to the common fold between the CaMKIIα AD and the dimeric yeast nuclear pore complex component NTF2^31,32^. The PF08332 MSA seeded an expanded sequence set for analysis of residue coevolution. The overlap of the evolution metrics with the complete AD dynamic network is shown in Fig. 3. The composite α_2–3_β_1–3_ contact network and the central β-sheet hinge were the principal drivers of CaMKII-AD coevolution. Coevolved contacts between helix β_1_ and the β sheet maintained the cross-section of the hydrophobic core. The long-range mechanical relays coupled the hydrogen-bonded hydrophobic core sidechains with β-sheet curvature^33^. The fluctuations in β-sheet curvature drove lateral intra-stack conformational spread via the Lat-Dim contacts.

### The evolution of the CaMKIIα AD targets the Lat-Dim contacts

**Figure 4:**
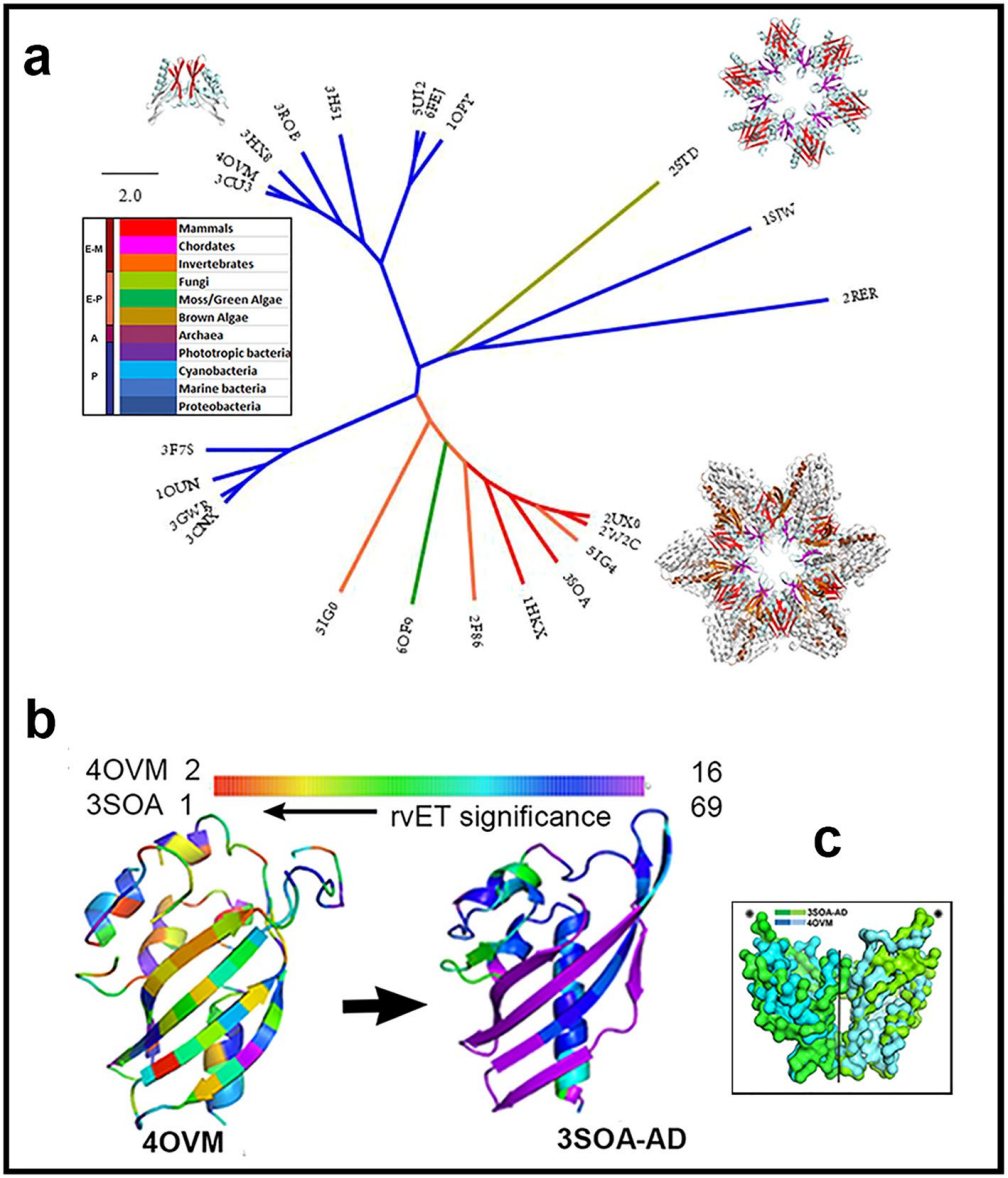
CaMKII phylogenetics (from 3D Structures). (**a**). Tree of 23 crystal structures of the CaMKII-AD homologs (color-coded by phyla) based on DALI scores. (**b**). The evolutionary trace (EV trace) for structures of an ancient bacterial enzyme (40VM.PDB) versus human CaMKIIα (3SOA.PDB). 3D Views in Supplementary Videos S9–S10. (**C**). Conservation of the dimer contact (RMSD = 0.69 nm). Source listed in Table S1.

I studied phylogeny to link structure to speciation, with the tree of life constructed from ribosomal RNA sequences as reference^34^. I superimposed all available crystal structures of the CaMKII-AD superfamily (n = 22), in addition to human CaMKIIα, to understand AD fold evolution. The phylogenetic tree (Fig. 4a) constructed from the DALI superimposition Z-scores demarcated prokaryotic and eukaryotic structures. The dimer was the dominant assembly (n = 11), followed by holoenzymes (n = 7) monomers (n = 2), a heterodimer. trimer and ring. Multiple superfamily members with different oligomeric states were found in *Streptomyces*, an ancient bacterial lineage^35^. The protozoan ring assemblies had similar architecture to the 3SOA AD hub, while the marine bacterium *Pirellula* sp.SH-Sr6A assembles a fourteen-subunit oligomer that may also form a homologous ring structure^33^.

The CaMKII-AD fold is a curved β-sheet (β_2_–β_6_) anchored to a long helix α_1_. The PF08332 MSA surface conservation profile identified the Vert-Dim β_2_–β_6_ interface as the most conserved. The 2D-heatmap revealed that the α_1_ N-terminus, loops at either end of helix α_3_, the β_4_–β_5_ loop, and β_5_ C-terminus were the variable elements. A global phylogenetic tree constructed from the PF08332 sequences related structural to organismal evolution. The tree representation was sufficient for this aim even though large evolutionary diversity in single-gene families is more accurately represented as a network^36^. The tree was demarcated into prokaryotic, eukaryotic, and archaeal clusters consistent with the phylogeny based on the crystal structures (Supplementary Fig. S2). The dimer has been recognized as an ancestral assembly module^33^, but its evolution based on sequence was not previously tracked from primordial bacteria to humans due to the low homology.

I used Evolutionary Trace (EV trace) to identify selection forces over short evolutionary timescales at two distant stages in the evolution of the superfamily. The EV trace was then mapped onto each structure. The ET MSAs were constructed from 20 4OVM.PDB (E value < 10^–2^) and nearly 500 3SOA.PDB (E value < 10^–60^) sequence homologs. For 3SOA.PDB, the KD DFG loop was the key determinant (rvET = 1.7 ± 1.0), followed by the R_286–305_ segment (rvET = 22.2 ± 1.7), both important for kinase activation. The rvET AD profiles revealed that in bacteria (4OVM.PDB) the fold underwent global evolution. In contrast, AD fold evolution in metazoan CaMKII holoenzymes (3SOA.PDB) was localized to the Lat-Dim contact rather than the Vert-Dim contact. Its rate was modest compared to KD evolution.

Finally, the structures of a *Streptomyces* enzyme (4OVM.PDB) and the human CaMKIIα AD dimer extracted from human CaMKIIα (3SOA.PDB) were superimposed (Fig. 4c). The CaMKIIα AD fold had elongated helix α_1_ and β-sheet segments relative to the bacterial enzyme that were utilized to form Lat-Dim contacts. The structural conservation of the Vert-Dim contact was indicated by the root mean square deviation (RMSD) between the common C^α^ backbone atoms of the superimposed structures (0.69 nm) and supported its sequence conservation deduced from the PF08332 MSA. I conclude that the Vert-Dim contact evolved most rapidly in bacteria, becoming fixed during metazoan evolution. The Lat-Dim contact was the focus of continuing evolution of the CaMKII AD fold in primates, in concert with KD evolution.

### The poikilotherm–homeotherm transition is a major step in CaMKIIα evolution

**Figure 5:**
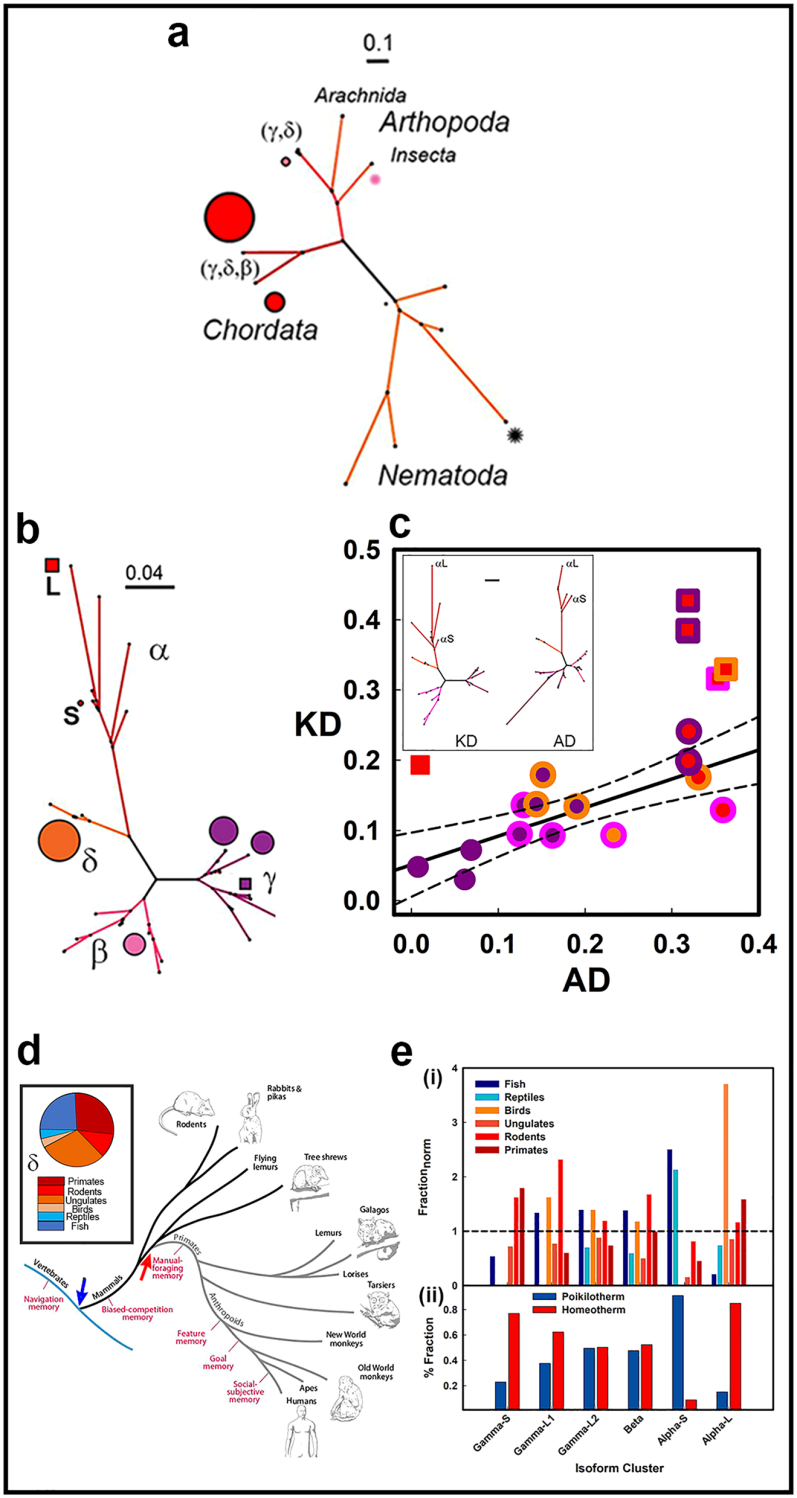
CaMKII phylogenetics (from 1D Sequences). (**a**). Metazoan CaMKII-AD evolution. Tree based on 1000 clustered homologs of the *C. elegans* CaMKII (black asterisk). Midge (pink asterisk). (**b**). Isoform evolution. Tree based on 2000 clustered homologs of the rat CaMKII α and β isoforms. Squares denote the most distant, larger CaMKIIα node (L) and the smallest y node with H:P ratios > $\overline{{{\text{H}}:{\text{P}}}}$ ± 2 s (1.5 ± 1.33), where $\overline{{{\text{H}}:{\text{P}}}}$ is the mean of the other nodes. Circles mark major clusters (diameter = membership; color = (i) phylum; (ii) isoform), Note difference in scale bar from that in Fig. S2C. (**c**) AD–KD coupling. The AD versus KD similarity plot compared the KD and AD phylogenetic trees color coded by isoform as in panel b (Box) The dual color symbols identify isoform pairs. The correlation coefficient, r = 0.59 with the best-fit (solid line) ± 95% confidence intervals (dashed lines). (**d**). Evolution of memory. Phylogenetic tree based on behavioral assays (from^39^). Arrows show the major bifurcations associated with the emergence of homeotherms (blue) and primates (red). Box: The distribution of the poikilotherms (blue, cyan) and homeotherms (rouge, red, orange, salmon) kingdoms in the sequences of the δ isoform. (**e**). Phylogenetic Species Diversity: Species distributions for major nodes of the α, β and γ isoforms based on kingdom (i) and homeostasis (ii). Source listed in Table S1.

The CaMKII holoenzyme structures were too few to trace the evolution of the CaMKIIα isoform, so I used CaMKII sequences instead. First, to understand the emergence of isoforms, I constructed phylogenetic trees from one thousand sequences most homologous to the *Caenorhabditis elegans* CaMKII, an ancient CaMKII with well-characterized structure and biochemistry (Fig. 5a). *C. elegans* lacks isoforms but has alternatively spliced variants. The nematodes (n = 18) formed the base of the stem that bifurcated to arthropod or chordate representatives. Insects (n = 8) and arachnids (n = 17) formed dedicated arthropod group nodes. Chordate as well as arthropod sequences segregated to a large mixed node (n = 106). The two chordate nodes (n = 554, 242) contained 271δ, 111γ and 7β isoform sequences with the β sequences all within the larger node. No sequences of the “α” isoform were found.

I, therefore, gathered one thousand closest homologs of the rat neuronal α and β isoforms to trace α isoform evolution. The resulting sequences (234α, 352β, 830γ, 575δ) were clustered (n = 34) for tree construction. This tree branched according to isoform rather than phyla (Fig. 5b) in contrast to the *C. elegans* rooted tree. This branching pattern extended to the individual domain tree topologies. The similarity score between the KD and AD trees was ~ 0.6 (Fig. 5c). This score was weaker but comparable to the scores (> 0.8) obtained for proteins with strongly interacting domains such as ribosomal components and the F_1_ ATP synthase subunits^37^. Thus, the domain coevolution, reflected in the similarity score is consistent with KD–AD interactions. In addition, the isoform dependent branching pattern shows that the variation of the domains within isoforms is less than between isoforms. This is also the case with the linkers where isoform conservation was more readily detected as the linker sequences are more variable^4^. I speculate that the conservation within isoforms and the domain coevolution may reflect adaptive selection for tissue-specific signal phospho-relays orchestrated by the KD^12^.

Analysis of the divergence and species composition of the major nodes of this tree provided insights into isoform evolution. The δ isoform was the least diverse with a single node. Its species composition is partitioned between poikilothermic (P) and homeothermic (H) vertebrates in ratio, H:P_δ_ = 2.24 (Fig. 5d). The species compositions of the other isoform nodes are shown in Fig. 5e. The major β isoform node was contaminated (~ 10%) with γ isoform sequences reflecting a close evolutionary relationship between these isoforms ($\overline{\Delta X}_{\beta - \delta } =$ 0.116 ± 0.008) (“Methods”(Phylogenetics)). The H:P ratios of the β (1.1) and the larger γ nodes (1.02, 1.66), but not the smallest γ node (γ_S_), were like δ. H:P_γS_ = 3.35. The α cluster was markedly different from the other isoform clusters. The smaller α node (α_S_) though most closely related to the other isoforms, nevertheless diverged significantly from them ($\overline{\Delta X}_{\alpha S - \beta \gamma \delta }$ = 0.208 ± 0.014 versus $\overline{\Delta X}_{\beta \gamma \delta }$ = 0.148 ± 0.009, $\overline{\Delta X}_{\gamma S - \beta \gamma \delta }$ = 0.084 ± 0.062). It consisted dominantly of poikilotherms (H:P_αS_ = 0.1). The larger node (α_L_) diverged even more $(\Delta X_{ \propto L - S} =$ 0.126). The α_L_ node consisted largely of mammalian particularly anthropoid sequences (H:P_αL_ = 5.66) consistent with the rapid evolution of memory in primates (Fig. 5d)^38^.

## Discussion

**Figure 6:**
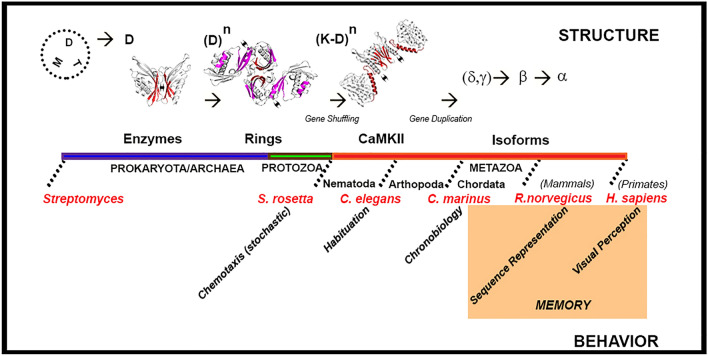
The correlation between the structural evolution of CaMKII and sensory behavior. Structure. The selection of the dimer (D) from other structures (M = monomer, T = tetramer) in bacteria seeded the emergence of ringed hub assemblies. The fusion with the kinase (K) domains coincided with the emergence of multicellularity (secondary structures color coded as in Fig. 1). Diversity was created by linker alternative splicing and enhanced by the generation of isoforms. Behavior. Work on model organisms suggests CaMKII evolution peaks with the development of cognitive memory. Advanced memory mechanisms (orange block). Source listed in Table S1.

This study has associated two dynamic processes that occur over dramatically different timescales—the macromolecular motions (< 10^−4^ s) of the assembly with the evolution of the CaMKII holoenzyme over billions of years (> 10^14^ s). The emergence of the CaMKII holoenzyme from ancient enzymes and the correlation with behavior involved distinct transitions coupled to fundamental changes in life forms (Fig. 6).

The structural phylogeny shows the CaMKII AD evolved via elongation and enhanced curvature of its β-sheet. The comparison of the *Streptomyces* and human CaMKIIα Vert-Dim dimers (Fig. 4c) supports spectroscopic^10,40^ and structural^41^ evidence that rings form by serial extension of vertically oriented dimer homologs. The elongated β-sheet topology assembles the hub and propagates conformational fluctuations across Lat-Dim as well as Vert-Dim contacts. The principal motions obtained from the PCA replicated the modes deduced from a survey of 743 PDB structures as fundamental collective motions driven by β-folds that propagate perpendicular to the plane of the fold^42^. What might be the role of these motions?

The KD–KD coupling mediated via the hub documented here is evidence for subunit cooperativity. CaMKII dimer assemblies have a lower Ca^2+^/CaM half-response dose (EC_50_) for kinase activation^10^, albeit similar Hill coefficients dominated by T286 trans-phosphorylation. The cooperativity can rationalize the different dimer and holoenzyme kinase activities. Holoenzyme structures obtained by cryo-electron tomography^43,44^, as opposed to the 3SOA.PDB crystal structure analyzed here, reveal that the inactive holoenzyme predominantly exists in an extended conformation without AD–KD contacts. The compliant, flexible linkers could act as mechanical low-pass filters to attenuate the KD–AD coupling, being wound in response to hub hinge motions. The hub interfacial (Vert-Dim, Lat-Dim) dynamics that orchestrate the overall KD–KD coupling should remain unaltered. The activated holoenzyme is also extended, but the R segments may interact with the hub^20^. The multimeric CaMKII architecture further allows multivalent binding to the actin cytoskeleton^45,46^ or multiple partners at the post-synaptic membrane in neurons^47^, and flexibility could be important for multivalent binding. Although models have utilized dimer and simpler geometries to reduce computational cost this study underscores the value of accurate models^48^ with interfacial energies constrained by structural data for modelling kinase activation.

This study documents that the α isoform has a unique evolutionary trajectory relative to the other isoforms. It emerged last with expression largely in the brain but evolved more rapidly than the rest. The link between CaMKII and sensory behavior required AD fusion with an ancestral KD after ring assemblies formed. Bacterial, archaeal, and eukaryotic serine-threonine kinases have a common ancestor^49,50^, while Ca^2+^/CaM dependent protein kinases are present in nitrogen-fixing bacteria^51^ so unicellular organisms could have provided the ancestral KD. Horizontal gene transfer-based shuffling events and the increased biosphere gene pool associated with the emergence of multicellular organisms presumably led to the fusion^52^. An ancestral CaMKII is found in the choanoflagellate *Salpingoeca rosetta* that alternates between unicellular and multicellular lifestyles, has a sensory response (chemotaxis), Homer and other primordial synaptic scaffolding proteins^53^. Alternative splicing, important in midge *Clunio marinus* chronobiology^15^ and short and long-term habituation in nematode *C. elegans* mechanosensory neurons^54^ preceded CaMKII isoforms, that were most likely generated by gene duplication events^12^. Advanced memory mechanisms from timed sequence representation in rodents (*Rattus norvegicus*) to visual memory and perception in humans (*Homo sapiens*) emerged in mammals^55^. These mechanisms required variable thresholds and expanded range of electrical, hence post-synaptic Ca^2+^, stimulation frequencies regulated by neuromodulators and associative learning^56,57^. Linker splicing could be too coarse a mechanism to execute such tasks without pathological consequences^8^. Furthermore, α has few spliced variants consistent with its limited tissue distribution^12^. The development of the hypothalamus for temperature homeostasis and the associated amygdaloid complex has been argued to have facilitated the evolution of advanced memory^38^. Strikingly, the rapid evolution of the α isoform followed the poikilotherm to homeotherm jump in contrast to the evolution of the other isoforms. Other physiological processes such as cardiovascular function are also affected in major ways. However δ, the isoform with the greatest similarity to α, is the major isoform expressed in the heart and has a more diverse splice variant and expression profile^12^. Furthermore, actin-binding capability important for heart function is the weakest for, and unlikely to exert selection pressure on, the α isoform^58^. Finally, the evolutionary trace mapped onto the CaMKIIα holoenzyme indicates that the evolution of the α isoform targets the Lat-Dim contact instrumental for conformational spread.

I, therefore, propose that conformational spread in the multimeric ring assembly tunes conformational transitions as in the bacterial flagellar motor^59^ to select and optimize the cooperative CaMKIIα kinase response to a broad range of Ca^2+^ pulse frequencies. The realization that a small αβ domain is partitioned into two distinct sectors based on the evolution of its residue contacts is a remarkable result that both explains the mechanics of the KD–KD coupling and rationalizes the maintenance of an energetically metastable, conformationally plastic sector. Evolution of the ancient Vert-Dim contact slowed after ring assemblies appeared allowing this contact to function as a conserved, semi-rigid connector module while the Lat-Dim contact formed a fine-grained dynamic code for KD–KD coupling with coevolved contacts that continue to change (Figs. 3, 4b). The code can be configured in two ways; by variation in the strength of the lateral interfacial contacts^60^ and/or by change of subunit stoichiometry^59^ mediated, in part, by activation-triggered subunit exchange^20,22^. The hub’s lateral sector contacts could be regulated by such exchange consistent with disassembly by R peptides^20^. The conformational fluctuations of the R segment, a major KD network node coupled to hub dynamics, will be modulated upon Ca^2+^/CaM binding^61^, subunit capture^9^ and substrate occupancy^62^. The formation of αβ heterooligomers, subunit stoichiometry variation, and KD–AD interactions could all enhance the combinatorial increase in the frequency range regulated by the hub.

## Methods

### Sequence analysis

The Pfam PF08332 MSA (1842 sequences) was input into ConSurf^63^ for estimation of residue conservation. Additional CaMKKI-AD superfamily sequences of isolates from diverse habitats and clinical repositories were added to PF08332 in GREMLIN (www.gremlin.org^64^). The expanded dataset (16,485 sequences (seq)) was submitted for MSA construction with HHblits (E < 10^–6^, 4 iterations, 75% coverage of the 3SOA AD (135 residues length (len)). Coevolution strength was given by the raw score (${S}_{r}$) a function of the pseudo-likelihood learning procedure, entropic correction, normalization (seq/len = 16,485/135) and separation between residue positions (> 3). The scaled score, ${S}_{s}$), is the normalized score (${S}_{r}/\overline{{S }_{r}})$. The top 30 couplings (${S}_{s}$ > 1.4 > 0.995 contact probability, (${P}_{c}$)), with validated contact, out of a total of 187 (${S}_{s}$> 0.5 > 0.265 ${P}_{c}$) were mapped on the 3D structure of the human holoenzyme. The 186 couplings represented 1% of the possible couplings between residue positions.

### Phylogenetics

The multiple sequence alignments (MSAs) of the CaMKII AD (PF08332) and the protein kinase domain (PF00069) were downloaded from the Pfam database (www.Pfam.org^65^). The thousand closely related homologs for each of the *C. elegans* CaMKII, rat CaMKIIα and CaMKIIβ sequences were identified with UniProt (www.UniProt.org^66^). The sequences were clustered with CD-Hit^67^ at the 0.8 cutoff threshold. Hierarchical clustering with the 0.8 cutoff, followed by a 0.6 cutoff was used for the PF08332, *C. elegans* and composite CaMKIIαβ sequence sets. The sequences were assigned to isoform either by direct readout of the UniProt headers or by comparison against the rat isoforms and selection by lowest E-value The MSAs of the cluster representatives were constructed with MUSCLE^68^. Crystal structures were downloaded from Protein Data Bank (www.rcsb.org^69^).

Unrooted trees were constructed with FastTree using the JTT model of amino acid evolution. Correlation matrices of paired tip distances ($Xi$,$Yi$) were constructed for each tree. Isoform diversity was estimated by the mean tip distance ($\overline{\Delta X} = \left( {\sum\nolimits_{i = 1}^{n} \Delta X/n} \right)$). The similarity between the CaMKII AD ($X$) and KD ($Y$) tree topologies was measured as the r score^37^.

1 $$r = \mathop \sum \limits_{i = 1}^{n} (Yi - \overline{Y})\left( {Xi - \overline{X}} \right)/\left\{ {\sqrt {\mathop \sum \limits_{i = 1}^{n} \left( {Yi - \overline{Y}} \right)^{2} } \sqrt {\mathop \sum \limits_{i = 1}^{n} \left( {Xi - \overline{X}} \right)^{2} } } \right\}$$

### Structure analysis

The topology of the 3D crystal structures was analyzed with CCP4^70^ and DALI^71^. The DALI C^α^–C^α^ distance correlation matrix alignment optimizes the correspondence between aligned residues pairs from multiple structures. The results were represented as a heatmap. The DALI scores Z_AB_, a metric for the correspondence between structures A and B corrected for the geometric mean length and the standard deviation, was used for the construction of pseudo-phylogenetic dendrograms.

The Evolutionary Trace traces the evolution of functional residues^72^. The homologous sequences for the bacterial AD (4OVM.PDB) and human CaMKIIα (3SOA.PDB) were downloaded, clustered, and used for dendrogram construction. Mutations localized at splits in the dendrogram identified possible functional sites. A contiguous patch of such residues identified a functional surface. The real value ET ($rvET$) score integrates the entropy and dendrogram location of each residue position in the MSA weighted for evolutionary distance.

2 $$rvET_{i} = 1 + \mathop \sum \limits_{n = 1}^{N = 1} w_{node} \left( n \right)\mathop \sum \limits_{g = 1}^{n} w_{group} \left( g \right)xs_{i}$$

where $w_{node}$ and $w_{group}$ are the phylogenetic tree nodes and tips, respectively. The $s_{i}$ is the information entropy that measures the frequency of occurrence, $\left( {f_{ia} } \right),$ of amino acid $a$ in residue position $i$ within the MSA.

3 $$s_{i} = - \mathop \sum \limits_{a = 1}^{20} f_{ia} ln\left( {f_{ia} } \right)$$

The frustration index, $${\Delta }Efr$$, computes the energies of the native residue contacts relative to the distribution of decoy energies, obtained by randomizing the identities of the residues in the native ($$ij)$$ contacts with $$n$$ randomly selected amino acid combinations ($$h$$)^73^.

4 $${\Delta }Efr_{ij} = \left( {{\Delta }E_{ij}^{N} - \overline{{{\Delta }E_{{i^{\prime } j^{\prime } }}^{D} }} } \right)/\sqrt {\left( {1/n} \right)\mathop \sum \limits_{k = 1}^{h} \left( {{\Delta }E_{{i^{\prime } j^{\prime } }}^{D} - \overline{{{\Delta e}_{{i^{\prime } j^{\prime } }}^{D} }} } \right)^{2} }$$

The native contact is “minimally frustrated” if its energy ${\Delta }E_{ij}^{ N}$ is at the lower end of the ${\Delta }E_{{i^{\prime } j^{\prime } }}^{D}$ decoy energy distribution (mean $\overline{{{\Delta }E_{ij}^{D} }}$). The contact is “highly-frustrated” if the converse is true. Contacts with an index higher than 0.78 and lower than − 1 were taken as minimally frustrated (“relaxed”) and highly frustrated (“stressed”), respectively. A case study of the integration of $rvET$ and ${{ \Delta }}Efr$ to understand protein design is available for calmodulin^74^.

### Protein dynamics

The monomer subunit A, the tetramer complex (subunits ACGI) and the ACGI tetramer AD human were extracted in silico from the human CaMKII holoenzyme structure (3SOA.PDB). The tetramer contained all lateral and vertical dimer contacts represented in the intact holoenzyme. Conformational ensembles of these structures were generated in Gromacs 4.5.7 (www.gromacs.org^75^) with tCONCOORD^27^ as described previously^76^. Full atom detail is preserved as a new structure is generated by random displacement of the constituent atoms within limits (2 nm^3^) followed by correction, up to 500 iterations, to eliminate bond violations. The solvent is implicit—solvent atoms are not simulated for increased computational speed, but a solvation parameter estimates the distance-dependent probability of a water molecule next to a particular atom for the prediction of unstable hydrogen bonds. This parameter was set to 2.2.

The essential collective motions were obtained by PCA^77^. The PCs were generated by diagonalization of the covariance matrix of C^α^ positions derived from the tCONCOORD ensembles. The variance of the PCs as given by the eigenvalues was taken as a measure of “motion”, with the first few PCs representing “slow” larger amplitude motions than those recorded by the later PCs on a relative timescale.

The conformer 3D structures were encoded as a 1D-string of four-residue fragments with a structural alphabet based on representative fragment states (letters) determined from frequently occurring conformations in 798 high-resolution X-ray structures^78^. The resulting array of 1D strings was used to derive a network of dynamic couplings based on normalized mutual information ($nMI$) with GSATools^79^). The correlation of conformational changes in a pair of protein segments *(i, j)* was calculated as normalized mutual information ($nMI$) between the associated columns in the structural string alignment.

5 $$nMI\left( {C_{i} ;C_{j} } \right) = \left( {I\left( {C_{i} ;C_{j} } \right) - \varepsilon \left( {C_{i} ;C_{j} } \right)} \right)/H_{{C_{i} C_{j} }}$$

where $C_{i}$ and $C_{j}$ are the relevant columns in the 1D string alignment, $I\left( {C_{i} ;C_{j} } \right)$ is the mutual information between them, $H_{{C_{i} C_{j} }}$ is the joint entropy, and $\varepsilon \left( {C_{i} ;C_{j} } \right)$ is the expected finite-size error. The 66 top couplings ($nMI$ > 0.15, fragment separation > 4 residues) were mapped on the 3D structures with a Pymol plugin^80^. Of these, 7 spanned the Vert-Dim interface, while 21 spanned the Lat-Dim interface.

The contribution of a node to the network scaled with its connectivity, estimated by the eigenvector centrality, *E*, calculated directly from the correlation matrix:

6 $$E.\;\left( M \right)_{{corr}} \; = \;E.\;{\uplambda }$$

where $\left( M \right)_{corr}$ is the correlation matrix and ${{ \uplambda }}$ is the corresponding eigenvalue.

Table S1 lists the software and algorithms used.

## Quantification and statistical analysis

### Phylogenetics and evolution

The local support used by FastTree, instead of traditional bootstrap values, is the estimation based on 1000 trials of the best probability of each split as assessed by the minimal evolution criterion^81^. The GREMLIN analysis of the HHblits AD MSA identified 186 coevolved couplings above the significance threshold (132.9 = (sequence number (16,485)/sequence length (131))^64^, of which the top 30 (> 0.995 significance) were mapped onto the crystal structure. Pearson’s coefficient was used to assess the similarity between KD and AD tree topologies.

### Dynamics

66,536 (16^4^) equilibrium conformations were generated for the monomer and tetramer structures extracted from 3SOA.PDB. The overlap between ensemble subsets was > 99% when subset size was < ¼ of the total ensemble, as reported previously for CaMKII KD structures^80^. The top network couplings mapped onto the AD crystal structure represented pairs above the 2s significance threshold in the distribution obtained after correction for the finite size error.

## Supplementary Information

Supplementary Information 1.

Supplementary Video 1.

Supplementary Video 2.

Supplementary Video 3.

Supplementary Video 4.

Supplementary Video 5.

Supplementary Video 6.

Supplementary Video 7.

Supplementary Video 8.

Supplementary Video 9.

Supplementary Video 10.

## Data Availability

The tree dendrograms, the GREMLIN job (ID 1592362472), subsets of the tCONCOORD ensembles, and the GSATools network files have been deposited in Mendeley (https://www.mendeley.com/reference-manager/library/collections/d81a4fb0-c1d5-4ee1-8a81-d31e0a34575d/). The PCA trajectories and structural models have been uploaded as Supplementary information.
