# Supplementary material for: Conformational spread drives the evolution of the calcium–calmodulin protein kinase II
Source: Sci Rep. 2022 May 19;12:8499. doi: 10.1038/s41598-022-12090-y (PMC9120016; doi:10.1038/s41598-022-12090-y)
Supplement: Supplementary file 1 — Supplementary Information 1. [file 41598_2022_12090_MOESM1_ESM.pdf]

Supplementary Information

TABLE S1: Software and Algorithms

All figures were composed in Photoshop from image files prepared with PyMOL or downloaded from online servers (DALI, EV-Trace, Frustratometer), and graphs prepared in Sigmaplot.

REAGENT or RESOURCE	SOURCE or CITATION	IDENTIFIER
CCP4	[70]	*www.ccp4.ac.uk/*
CD-Hit	[67]	*weizhongli-lab.org/cd-hit*
Cytoscape 3.7	-	*cytoscape.org*
DALI	[71]	*ekhidna2.biocenter.helsinki.fi/dali*
EV-Trace	[72]	*evolution.lichtargelab.org*
FastTree	[81]	*www.microbesonline.org/fasttree/*
FigTree 1.4	-	*evomics.org/resources/software/molecular-evolution-software/figtree*
Frustratometer	[73]	*frustratometer.qb.fcen.uba.ar*
GREMLIN	[64]	*www.gremlin.org*
Gromacs	[75]	*www.gromacs.org*
GSATools	[79]	*pandinilab.org/gsatools.html*
MUSCLE	[82]	*www.drive5.com/muscle/*
Pfam	[65]	*www.Pfam.org*
Photoshop CS6		*www.adobe.com//photoshop*
Protein Data Bank	[69]	*www.rcsb.org*
PyMOL 1.74		*www.pymol.org*
Sigmaplot 12.0		*www.sigmaplot.co.uk/sigmaplot/*
tCONCOORD	[27] -	*www3.mpibpc.mpg.de/groups/de_groot/dseelig/tconcoord.html*
UniProt	[66]	*www.UniProt.org*
VMD 1.91		*www.ks.uiuc.edu/Research/vmd/*

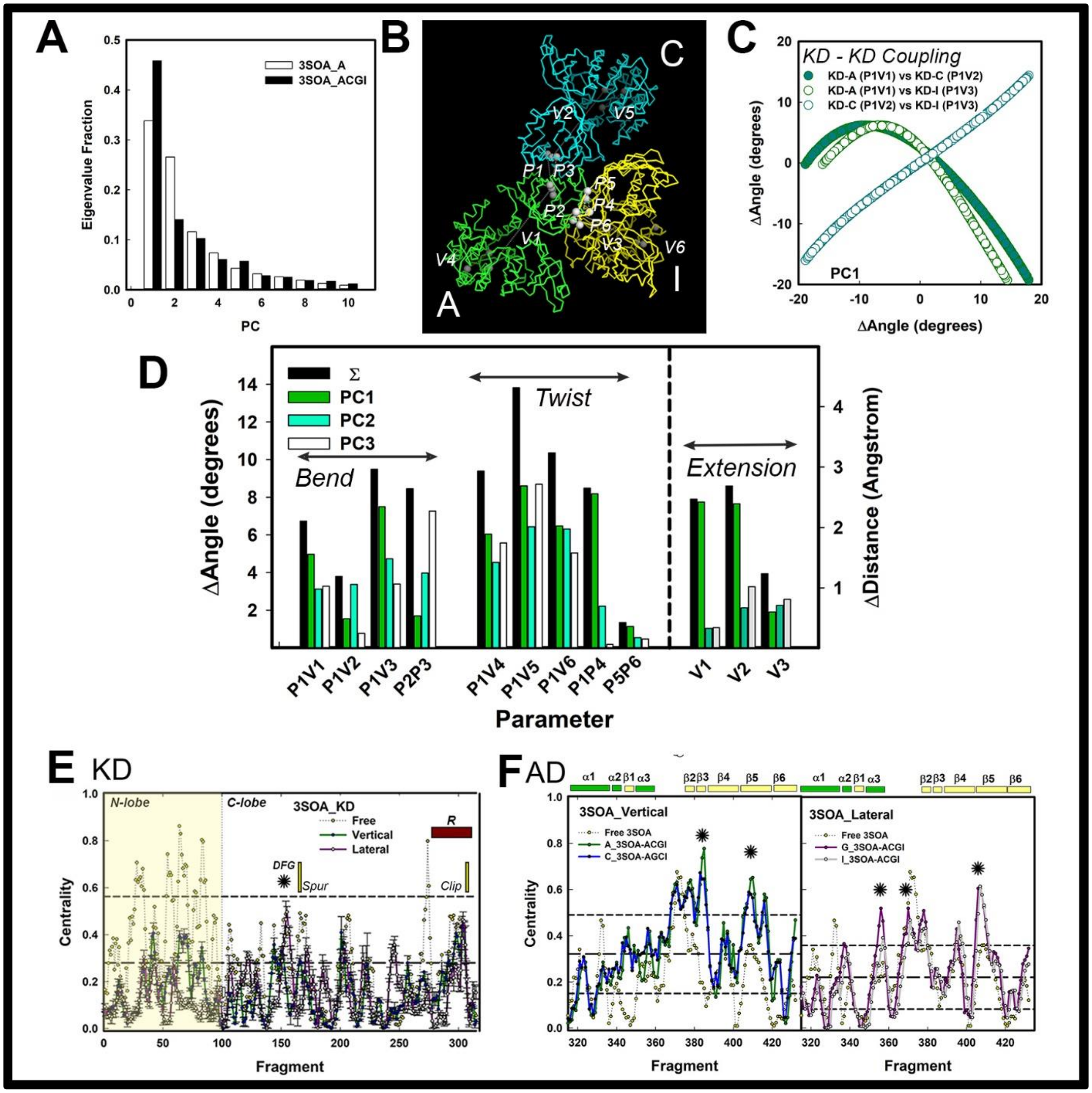

Fig. S1. (A-D) Principal Component Analysis. A. *The normalized PC1-10 monomer (3SOA-A) and tetramer (3SOA-ACGI) eigenvalue distributions. PCs 1-3 accounted for 70% of the total (n = 5,256) eigenvalue amplitude. The comparison of PCs 1-2 shows that tetramerization increases motion anisotropy.* ***B.*** *The vectors (V1-V6) and planes (P1-P5) for measurement of collective PC motions mapped on the structures of 3SOA.pdb subunits A, C, and I. Plane orientations are given by vectors perpendicular to them. The GROMACS gmx angle operator measured vector-vector angular dispersions. The gmx distance operator measured vector length fluctuations. The Vert-Dim AC interface is defined by inter-domain P1 and intra-domain P2 (A) and P3 (C). The Lat-Dim AI interface is described by inter-domain P4 and intra-domain P5 (A) and P6 (I). The perpendicular vector pairs to P2P3 and P5P6 measured hinge motions. The V1P1, V2P1 and V3P1 pairs reported A, C and I bending respectively. The V4P1, V5P1 and V6P1 reported A, C and I twist respectively. P1P4 reported interfacial Vert – Lat AD coupling. V1, V2 and V3 monitored*

radial movements of the A, C, and I KDs respectively out from the ADs. ***C.*** *The coupling between KD motions measured as the angular dispersion of the PC motions.* ***D.*** *The contribution of the PC1 – PC3 to the angular dispersions between the selected vector pairs.* ***(E-F) Network construction E.*** *Monomer and tetramer-KD network centrality plot partitioned into (A, C) and (G, I) AD subsets. Mean (dashed line) ± standard deviation (short-dashed lines) for the monomer profile. Asterisks mark central tetramer network nodes. KD-AD contacts (yellow bars).* ***F.*** *Tetramer-AD network centrality plot partitioned and marked with asterisks as in E.* ***Source listed in Table S1.***

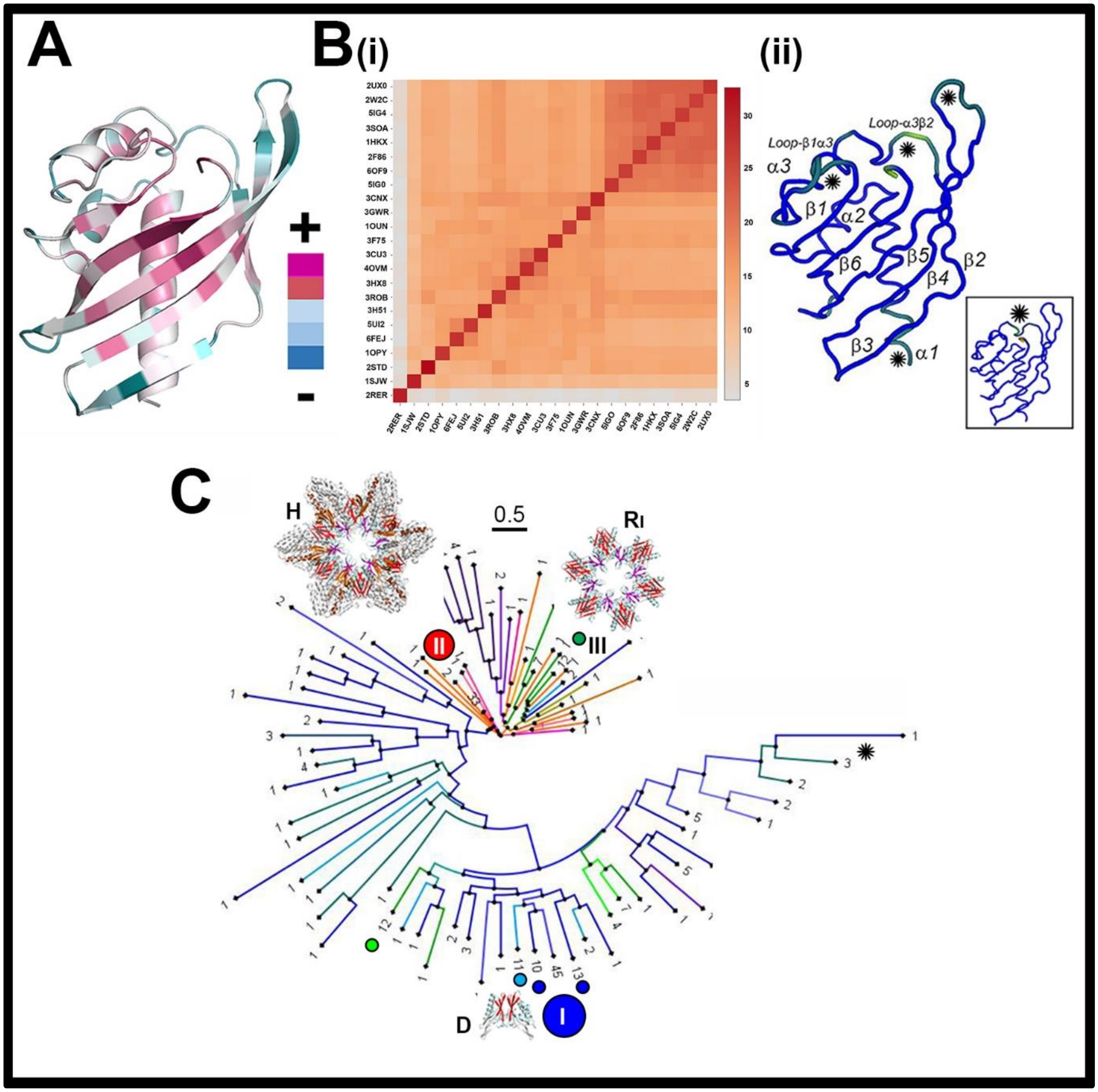

Fig. S2: (A-B) Structural conservation in the CaMKII-AD. A. *CONSURF residue conservation (MSA = 1842 sequences).* ***B. (i)*** *Heatmap of the DALI alignment of the human CaMKIIα AD (PDB:3SOA) with 22 structural homologs.* ***.*** *Bar = color-coded DALI Z scores.* ***(ii)*** *Superposition of the 23 structures Asterisks indicate variable segments (index: blue (low) -> orange (high)).* ***Box****: DALI alignment of the six CaMKII holoenzyme structures. Variation is restricted to the N-terminal segment of the α3-β2 loop and the β6 C-terminus (asterisk).* ***C. CaMKII AD superfamily tree.*** *The tree was color coded following Fig. 4A. It was constructed from 81 representative sequences of clusters obtained after CD-Hit compaction of PF08332. The major nodes are composed of bacteria (I (P. n =45)), vertebrate (II (E-M. n = 33)) and diatom/fungal (III (E-P. n =)) clusters, followed by four smaller (3 bacterial and a fungal node in a large group that includes node-1. The AD assemblies associated with nodes I-III (D (dimer, β contact (red)), Ri (ring, lateral β contact (magenta)), H (holoenzyme. (R (brown), KD C-lobe (orange))) are shown. The asterisk indicates a possible common ancestor. The examples of horizontal gene transfer (HGT) included a marine bacterium in node*

III and protozoan fungal and green algae around node I. These examples notwithstanding, the tree linked ancient extremophiles and other bacterial species to mammalian and archaeal relatives. The most populous node (I) contained enzymes from extremophilic nitrate and sulfur-reducing bacteria for aromatic compound biosynthesis. Its long branch lengths indicated a high rate of evolution. The second most populous node (II) was composed of metazoan sequences represented by the rat CaMKIIβ AD. It's short branch lengths indicated constrained evolution consistent with integration into multiple phosphorylation pathways following KD acquisition. Protozoan sequences formed the third most populous node (III (n =18)). Source listed in ***Table S1.***

Video S1. Related to Fig. 1a-b. *The tetramer orientations in Figs. 1 a and 1 b related by 90° rotation.*

Video S2. Related to Fig. 1c. *Filtered PC, PC1, PC2, PC3 trajectories for the 3SOA tetramer (subunits A (green), C (cyan), G (purple), I (white)).*

Video S3. Related to Fig. 2c. *Filtered PC, PC1, PC2, PC3 trajectories for the 3SOA tetramer AD hub (subunits A (green), C (cyan), G (purple), I (white)).*

Video S4. 3D representation of Fig. 2a*.*

Video S5. 3D representation of Fig. 2b*.*

Video S6. 3D representation of Fig. 2c*.*

Video S7. 3D representation of Fig. 2d*.*

Video S8. 3D representation of Fig. 3*.*

Video S9. 3D representation of Fig. 4b.4ovm

Video S10. 3D representation of Fig. 4b.3soa*. KD + AD.*
